# Psychological impact of COVID-19 pandemic on healthcare workers in a highly burdened area of north-east Italy

**DOI:** 10.1017/S2045796020001158

**Published:** 2019-12-17

**Authors:** A. Lasalvia, C. Bonetto, S. Porru, A. Carta, S. Tardivo, C. Bovo, M. Ruggeri, F. Amaddeo

**Affiliations:** 1 UOC Psichiatria, Azienda Ospedaliera Universitaria Integrata di Verona, Verona, Italy; 2 Section of Psychiatry, Department of Neuroscience, Biomedicine and Movement Sciences, University of Verona, Verona, Italy; 3 Section of Occupational Medicine, Department of Diagnostics and Public Health, University of Verona and Clinical Unit of Occupational Medicine, Integrated University Hospital of Verona, Italy; 4 Section of Hygiene, Department of Diagnostic and Public Health, University of Verona, Italy; 5 Health Director, Azienda Ospedaliera Universitaria Integrata di Verona, Verona, Italy; 6 UOC Psicosomatica e Psicologia Medica, Azienda Ospedaliera Universitaria Integrata di Verona, Verona, Italy

**Keywords:** Anxiety, COVID-19, depression, health workers, mental health, post-traumatic stress

## Abstract

**Aims:**

Healthcare workers exposed to coronavirus 2019 (COVID-19) patients could be psychologically distressed. This study aims to assess the magnitude of psychological distress and associated factors among hospital staff during the COVID-19 pandemic in a large tertiary hospital located in north-east Italy.

**Methods:**

All healthcare and administrative staff working in the Verona University Hospital (Veneto, Italy) during the COVID-19 pandemic were asked to complete a web-based survey from 21 April to 6 May 2020. Symptoms of post-traumatic distress, anxiety and depression were assessed, respectively, using the Impact of Event Scale (IES-R), the Self-rating Anxiety Scale (SAS) and the Patient Health Questionnaire (PHQ-9). Personal socio-demographic information and job characteristics were also collected, including gender, age, living condition, having pre-existing psychological problems, occupation, length of working experience, hospital unit (ICUs and sub-intensive COVID-19 units vs. non-COVID-19 units). A multivariable logistic regression analysis was performed to identify factors associated with each of the three mental health outcomes.

**Results:**

A total of 2195 healthcare workers (36.9% of the overall hospital staff) participated in the study. Of the participants, 35.7% were nurses, 24.3% other healthcare staff, 16.4% residents, 13.9% physicians and 9.7% administrative staff. Nine per cent of healthcare staff worked in ICUs, 8% in sub-intensive COVID-19 units and 7.6% in other front-line services, while the remaining staff worked in hospital units not directly engaged with COVID-19 patients. Overall, 63.2% of participants reported COVID-related traumatic experiences at work and 53.8% (95% CI 51.0%–56.6%) showed symptoms of post-traumatic distress; moreover, 50.1% (95% CI 47.9%–52.3%) showed symptoms of clinically relevant anxiety and 26.6% (95% CI 24.7%–28.5%) symptoms of at least moderate depression. Multivariable logistic regressions showed that women, nurses, healthcare workers directly engaged with COVID-19 patients and those with pre-existing psychological problems were at increased risk of psychopathological consequences of the pandemic.

**Conclusions:**

The psychological impact of the COVID-19 pandemic on healthcare staff working in a highly burdened geographical of north-east Italy is relevant and to some extent greater than that reported in China. The study provides solid grounds to elaborate and implement interventions pertaining to psychology and occupational health.

## Introduction

Italy was the first western country to be affected by the coronavirus-2019 (COVID-19) pandemic. The first autochthonous COVID-19 case was diagnosed in the town of Codogno (Lombardy), on 18 February. The first Italian death resulting from COVID-19 occurred on 21 February and was a resident of the municipality of Vò Euganeo, a small town near Padua (Veneto). On 24 February, the Italian government established two ‘red zones’ in Codogno and Vò Euganeo. On 8 March, the government decided to extend these extraordinary measures to all of Lombardy, Veneto and some neighbouring provinces of Emilia-Romagna. Eventually, a nationwide lockdown was established on 11 March (Italian Ministry of Health, 2020).

The exponential rise of COVID-19 cases and the increasingly urgent need for intensive care unit surge capacity for the management of critically ill patients posed an extraordinary strain on the healthcare systems of Lombardy, Veneto and Emilia-Romagna (Lazzerini and Putoto, 2020; Remuzzi and Remuzzi, 2020). Starting from mid-March 2020, activities within hospitals in the most affected regions underwent a rapid and profound re-organisation (Faccincani *et al*., 2020; Marcon *et al*., 2020).

Under such circumstances, healthcare staff in the affected regions were experiencing heavy workload conditions at a high risk of infection (Boccia *et al*., 2020). As of 6 April, 9% of infections (i.e. 12.681 cases on 124.527 overall cases) had occurred among healthcare personnel (ISS, 2020), leading to further loss of capacity for hospitals to respond. Hospital overcrowding, together with lack of adequate respiratory protective devices, were the main factors contributing to the high infection rate of medical personnel during the first weeks of the pandemic (Anelli *et al*., 2020).

Moreover, healthcare professionals working in close contact with COVID-19 patients are made vulnerable to adverse mental health consequences. Research conducted during past epidemics found that increased workload, fear of infection, frustration, physical exhaustion and inadequate personal equipment had a substantial impact on the mental health of healthcare workers (Salazar de Pablo *et al*., 2020), with staff in contact with affected patients showing greater levels of both acute or post-traumatic stress and psychological distress compared with lower risk controls (Kisely *et al*., 2020). A number of papers published over the last few months confirmed that a considerable proportion of healthcare workers within secondary and tertiary hospitals developed adverse psychological outcomes also during the COVID-19 pandemic (Pappa *et al*., 2020). These studies found that front-line workers and those with the most direct contact with COVID-19 patients are more at risk for developing mental health symptoms (Guo *et al*., 2020; Lai *et al*., 2020; Lu *et al*., 2020; Zhang *et al*., 2020a, b; Zhu *et al*., 2020).

Most literature published on this topic was carried out in China or other Asian countries (Pappa *et al*., 2020; Sanghera *et al*., 2020; Song *et al*., 2020). The generalisability of findings to other parts of the world is therefore limited by the fact that healthcare systems vary greatly and, consequently, their response to the current COVID-19 pandemic.

Like many countries, the Italian healthcare system was ill-prepared to tackle an emergency of such magnitude. Italy, being the first nation in the western world to be affected by the pandemic, had no time to arrange a rapid and effective response to the spread of the virus and had no previous experience on how to handle a pandemic of this scale. Indeed, Italian healthcare professionals had never faced an epidemic before and were not sufficiently trained on how to apply and follow appropriate infection control practices and procedures.

The current study aimed to assess mental health outcomes among personnel working in the Verona University Hospital during the lockdown phase of the COVID-19 pandemic by evaluating the presence and magnitude of symptoms of post-traumatic distress, anxiety and depression, and by analysing associated risk factors.

## Methods

### Study design

The type of research applied in this study is explanatory in nature. This study represents the baseline evaluation of a larger longitudinal project jointly launched by the Verona University Hospital Trust and the Section of Psychiatry at the University of Verona. The longitudinal study will assess the psychological impact of the COVID-19 pandemic on hospital workers during the lockdown period, after two months, and at one year. Baseline data collection was carried out between 21 April and 6 May 2020 using a web-based questionnaire hosted on the survey platform ‘SurveyMonkey’. The online survey required about 15–20 min to be completed. At the baseline evaluation, each participant was asked to generate a password that should have been used also at the subsequent follow-ups; this was required for the research team to longitudinally link the questionnaires completed by a given hospital worker at each evaluation point. The study description and the invitation to participate as well as the link to the online questionnaire was published in the hospital's newsletter and sent via e-mail to all hospital workers by the trust administration. A reminder for completing the questionnaire was sent around after one week. The survey was anonymous, and confidentiality of information was granted. The survey was approved by the Ethics Committee of the Provinces of Verona and Rovigo (approval No. 22002; 17 April 2020).

### Setting and participants

The Verona University Hospital is the second-largest hospital trust in Italy in terms of bed quantity and the fifth largest in terms of admissions. The hospital staff is composed of 5942 personnel (including nearly 1200 residents of the medical specialty schools at the University of Verona). Beginning 17 March 2020, the Veneto regional government converted part of the hospital into a ‘COVID-19 hospital’. Thus, dedicated pathways for both suspected and confirmed COVID-19 cases were established within the hospital, as well as in other hospital units located in clearly restricted areas specifically devoted to the treatment of COVID-19 patients. All staff working in the Verona University Hospital during the lockdown phase of the pandemic were asked to participate in the study.

### Assessment measures

The psychological distress was assessed using the Impact of Event Scale-Revised (IES-R) (Weiss and Marmar, 1997; Craparo *et al*., 2013), a 22-item self-report that measures subjective distress caused by traumatic events on a 5-point scale from 0 (not at all) to 4 (extremely) during the previous seven days. The IES-R was completed only by those who had experienced stressful/traumatic events at work related to COVID-19. Participants were first asked to identify a stressful life event that they may have encountered at work and to specify which kind of stressful event it was. Respondents were then asked to rate how much they were distressed or bothered during the past seven days by each item listed in the IES-R. The maximum score is 88 (worst post-traumatic stress state). We used a cut-off score of 24 for detecting symptoms of post-traumatic distress that deserve clinical attention (Creamer *et al*., 2003).

Symptoms of anxiety were assessed by the Self-rating Anxiety Scale (SAS) (Zung, 1971, 1980) that contains 20 items, each rated on a 5-point scale from 1 (a little of the time) to 4 (most of the time). The maximum score of 80 indicates an extremely high anxiety level and the cut-off score for clinically significant anxiety symptoms is 36 (Dunstan and Scott, 2020).

Symptoms of depression were assessed by the Patient Health Questionnaire (PHQ-9) (Kroenke *et al*., 2001), a self-rated 9-item scale that asks if the subject has experienced symptoms of depression in the previous two weeks. Subjects are asked to rate how often each symptom occurred: 0 (not at all), 1 (several days), 2 (more than half the days), or 3 (nearly every day). Total PHQ-9 scores range from 0 (absence of depressive symptoms) to 27 (most severe depressive symptoms). We used a cut-off score of 10 to indicate a condition that potentially deserves clinical attention (Kroenke and Spitzer, 2002).

Two ad hoc instruments exploring perceived job stress and perception of risk, modified, respectively, from Imai *et al*. (2005) and Maunder *et al*. (2006), were administered.

Personal socio-demographic information and job characteristics were also collected, including gender, age, living condition, having psychological problems developed before the COVID-19 outbreak requiring specialised help, occupation, length of working experience, place of work (hospital unit). For the purpose of analysis, the various hospital units were grouped according to the degree of clinical engagement with COVID-19 patients, from most engaged to least engaged: Intensive Care Units (that during the lockdown phase were entirely dedicated to critically ill COVID-19 patients), sub-intensive COVID wards (i.e. infectious disease, pulmonary medicine and internal medicine wards specifically dedicated to COVID-19), frontline services dealing with COVID patients (i.e. radiology and emergency department), non-COVID wards, laboratory diagnostic services (i.e. laboratory medicine, transfusion medicine, immunology, pathology, microbiology) and administration.

### Statistical analysis

Statistical analyses were carried out using SPSS 22 and Stata 15. Descriptive statistics were reported in frequencies and percentages. Comparisons between categorical variables were performed by Chi-square or Fisher's exact tests where appropriate.

The precision of the proportion estimate for each adverse outcome was determined by calculating the margin of error for the two-sided 95% confidence interval (95%CI).

The association between each adverse outcome (post-traumatic distress, anxiety and depression) and each potential risk factor selected *a priori*, on clinical or empirical grounds and derived from the relevant literature (Kisely *et al*., 2020; Pappa *et al*., 2020) was explored by estimating unadjusted odds ratios (ORs) and 95%CIs using univariable logistic regression models. Subsequently, multivariable logistic regression models for the same outcomes gave adjusted ORs and 95%CIs. Goodness-of-fit measures were estimated for these models.

In a secondary analysis, missing data on outcomes were estimated using a multiple imputation approach based on logistic regression (‘mi impute logit’ Stata command).

A sensitivity analysis using an alternative modelling strategy, that is by considering the outcomes in terms of continuous scores and estimating linear regression models, was performed.

The alpha level was set to 0.05 for all effects.

## Results

### Personal and job characteristics

|Overall, 2195 workers, corresponding to 36.9% of the eligible population, completed the on-line survey.

The representativeness of participants was assessed by comparing two key characteristics found to be associated with response/non-response pattern for all the three outcome domains and for which official statistics from the Verona University Hospital were available (i.e. occupation and exposure to COVID-19 patients). For details, see on-line supplementary part 1. Overall, the study sample overlapped with the Verona University Hospital staff both in terms of occupational profile and percentage of healthcare workers employed in units directly engaged with COVID-19 patients, thus indicating that the sample addressed here is representative of the eligible population.

Table 1 shows personal and job characteristics of participants.

**Table 1.** Personal and job characteristics of participants (*n* = 2195)

	*n*	%
**Gender** (9 missing)		
Male	539	24·7
Female	1647	75·3
**Age** (9 missing)		
<36 yrs	702	32·1
36–55 yrs	1102	50·4
>55 yrs	382	17·5
**Living condition** (7 missing)		
Alone	358	16·4
With family/other relatives	1830	83·6
**Occupation**		
Physicians	306	13·9
Residents	361	16·4
Nurses	783	35·7
Other healthcare staff	533	24·3
Administrative staff	212	9·7
**Length of working experience** (14 missing)		
<6 yrs	668	30·6
6–20 yrs	670	30·7
>20 yrs	843	38·7
**Work place** (39 missing)		
Intensive care units	195	9·0
Sub-intensive care wards for COVID-19 pts^a^	178	8·3
Frontline services dealing with COVID-19 pts^b^	164	7·6
Non-COVID wards	1199	55·6
Laboratory diagnostic services^c^	238	11·0
Administration	182	8·4
**Having pre-existing psychological problems**		
Yes	135	6·2
No	2060	93·8

a Infectious Disease Unit, Pulmonary Medicine, Internal Medicine units converted specifically to COVID-19.

b Radiology and Emergency Department.

c Laboratory Medicine, Transfusion Medicine, Immunology, Pathology, Microbiology.

### Levels of job stress and perception of risk

Table 2 (upper part) shows percentages of healthcare workers reporting some kind of job-related stress experienced during the COVID-19 pandemic. The vast majority of participants reported more stress at work; moreover, most participants reported more conflict among colleagues, increased workload and additional tasks that they were not responsible for pre-COVID.

**Table 2.** Job stress and perception of risk in the overall sample (*n* = 2195)

	*n*	%
**JOB STRESS** (34 missing)		
**There was more conflict among colleagues**		
Yes	1091	50·5
No	494	22·9
As usual	576	26·7
**I felt more stressed at work**		
Yes	1847	85·5
No	120	5·6
As usual	194	9·0
**I had to do work that I usually don't do**		
Yes	1272	58·9
No	889	41·1
**I had an increased workload**		
Yes	1415	65·5
No	746	34·5
**PERCEPTION OF RISK** (34 missing)		
**I should not be looking after patients with COVID-19** (796 subjects do not look after COVID-19 pts)		
No	900	65·9
Yes	465	34·1
**I accept the risk of getting COVID-19 as part of my job** (566 subject do not face with COVID-19 pts)		
No	158	9·9
Yes	1437	90·1
**I'm afraid of getting ill with COVID-19**		
No	300	13·9
Yes	1790	82·8
Don't know	71	3·3

Table 2 (lower part) reports the perception of risk. The vast majority of healthcare workers were afraid of falling ill with COVID-19; however, they considered the risk of infection as part of their job. When stratifying levels of job stress and perception of risk by place of work, a significantly higher percentage of staff reporting more conflict among colleagues, increased workload, unusual additional tasks and greater fear of infection was found among those working in ICUs and in sub-intensive care wards for COVID-19 than in other hospital units; moreover, when stratifying by occupational profile, a higher percentage of nurses reported greater job stress and perception of risk than other hospital workers (see the on-line supplementary part 2).

### Post-traumatic distress, anxiety and depression

The IES-R, the SAS and the PHQ-9 were completed, respectively, by 91.3%, 90.7% and 90.2% of eligible participants. When comparing percentages of completers and non-completers with respect to personal information across the three outcome measures, no staff characteristic was significantly associated with the pattern of response/no response, suggesting that the sample addressed here was not biased in terms of completion of instruments. Differences between completers and non-completers are detailed in the on-line supplementary part 3.

Overall, 63.2% reported having experienced some traumatic events related to COVID-19. The percentages of those who reported COVID-related traumatic experiences significantly differed by occupational profile (nurses 74%; other healthcare workers 63%; physicians 59%; residents 56%; administrative staff 41%; *p* < 0·001) and place of work (ICUs 89%; sub-intensive COVID units 90%; other frontline services 77%; no COVID wards 59%; laboratory services 49%; administration 45%; *p* < 0·001), with nurses and healthcare staff working in both ICUs and sub-intensive COVID units reporting more traumatic events. The frequency distribution of specific traumatic events is given in the on-line supplementary part 4. In brief, the most frequent traumatic themes were related to the fear of infection, demanding work conditions and dealing with the death and dying, reported, respectively, by 28.7%, 27.4% and 15.4% of respondents.

Among those who reported a COVID-related traumatic experience, 53.8% (95% CI 51·0%–56.6%; margin of error 2.8%) showed clinically relevant symptoms of post-traumatic distress. Moreover, in the overall sample, 50.1% (95% CI 47·9%–52.3%; margin of error 2.2%) reported symptoms of clinically significant anxiety and 26.6% (95% CI 24.7%–28.5%; margin of error 1.9%) symptoms of at least moderate depression.

As shown in Table 3, stratifying by personal and job characteristics, the proportion of participants scoring above the cut-off points in the three outcome scales widely differed.

**Table 3.** Distribution of risk factors across outcomes (post-traumatic distress, anxiety, depression) in the overall sample (*n* = 2195)

	**Post-traumatic distress** (119 missing)		**Anxiety** (204 missing)		**Depression** (216 missing)	
	**<24 IES-R** *n* (%)	**⩾24 IES-R** *n* (%)	**<36 SAS** *n* (%)	**⩾36 SAS** *n* (%)	**<10 PHQ** *n* (%)	**⩾10 PHQ** *n* (%)
**Number of subjects (%)**	574 (46·2%)	668 (53·8%)	993 (49·9%)	998 (50·1%)	1452 (73·4%)	527 (26·6%)
**Gender**						
Male	159 (55·2)	129 (44·8)	334 (66·8)	166 (33·2)	407 (82·1)	89 (17·9)
Female	413 (43·4)	538 (56·6)	653 (44·0)	831 (56·0)	1039 (70·4)	437 (29·6)
**Living condition**						
Alone	95 (41·7)	133 (58·3)	160 (48·6)	169 (51·4)	206 (63·6)	118 (36·4)
With family/other relatives	478 (47·3)	532 (52·7)	828 (50·0)	828 (50·0)	1240 (75·2)	409 (24·8)
**Occupation**						
Physicians	97 (57·4)	72 (42·6)	193 (66·6)	97 (33·4)	238 (82·4)	51 (17·6)
Residents	121 (63·7)	69 (36·3)	207 (61·2)	131 (38·8)	254 (75·6)	82 (24·4)
Nurses	181 (35·2)	333 (64·8)	260 (37·2)	438 (62·8)	468 (67·3)	227 (32·7)
Other healthcare staff	138 (46·5)	159 (53·5)	228 (48·2)	245 (51·8)	342 (72·8)	128 (27·2)
Administrative staff	37 (51·4)	35 (48·6)	105 (54·7)	87 (45·3)	150 (79·4)	39 (20·6)
**Work place**						
Intensive care units	56 (34·4)	107 (65·6)	67 (36·4)	117 (63·6)	107 (58·5)	76 (41·5)
Sub-intensive care wards for COVID-19 pts^a^	53 (24·9)	99 (65·1)	64 (38·6)	102 (61·4)	106 (63·9)	60 (36·1)
Services dealing with COVID-19 pts^b^	57 (47·9)	62 (52·1)	72 (48·3)	77 (51·7)	106 (72·6)	40 (27·4)
Non-COVID wards	309 (50·0)	309 (50·0)	566 (52·7)	509 (47·3)	811 (75·7)	260 (24·3)
Laboratory diagnostic services^c^	46 (44·2)	58 (55·8)	117 (52·9)	104 (47·1)	166 (75·5)	54 (24·5)
Administration	44 (64·7)	24 (35·3)	90 (55·2)	73 (44·8)	131 (81·9)	29 (18·1)
**Length of working experience** (yrs)						
<6	205 (53·7)	177 (46·3)	324 (53·6)	281 (46·4)	443 (73·7)	158 (26·3)
6–20	186 (47·1)	209 (52·9)	289 (47·9)	314 (52·1)	430 (72·3)	165 (27·7)
>20	180 (39·4)	277 (60·6)	373 (48·4)	397 (51·6)	568 (73·8)	202 (26·2)
**Having pre-existing psychological problems**						
Yes	19 (23·5)	62 (76·5)	37 (29·8)	87 (70·2)	65 (52·4)	59 (47·6)
No	555 (47·8)	606 (52·2)	956 (51·2)	911 (48·8)	1387 (74·8)	468 (25·2)
**Experienced traumatic event**						
Yes	574 (46·2)	668 (53·8)	472 (38·8)	745 (61·2)	796 (65·7)	415 (34·3)
No	-	-	521 (67·3)	253 (32·7)	656 (85·4)	112 (14·6)
**Afraid of getting ill with COVID-19**						
No	75 (66·4)	38 (33·6)	212 (74·9)	71 (25·1)	234 (83·9)	45 (16·1)
Yes	480 (43·9)	613 (56·1)	745 (45·3)	899 (54·7)	1169 (71·4)	468 (28·6)
Don't know	19 (52·8)	17 (47·2)	36 (56·3)	28 (43·8)	49 (77·8)	14 (22·2)

a Infectious Disease Unit, Pulmonary Medicine, Internal Medicine units converted specifically to COVID-19;.

b Radiology and Emergency Department;.

c Laboratory Medicine, Transfusion Medicine, Immunology, Pathology, Microbiology.

Overall, women, nurses and staff working in ICUs or sub-intensive COVID units reported higher percentages of post-traumatic distress, anxiety and depression. Specifically, 57% of women reported a severe level of post-traumatic distress, 56% displayed severe anxiety and 30% severe depression. In addition, 65% of nurses reported severe post-traumatic symptoms, 63% showed severe anxiety and 33% severe depression. Healthcare workers in ICUs reported symptoms of post-traumatic distress in 66% of cases, 64% severe anxiety and 42% severe depression; similarly, staff working in sub-intensive COVID units reported percentages of post-traumatic distress, anxiety and depression, respectively, in 65%, 61.4% and 36% of cases.

**Fig. 1.**
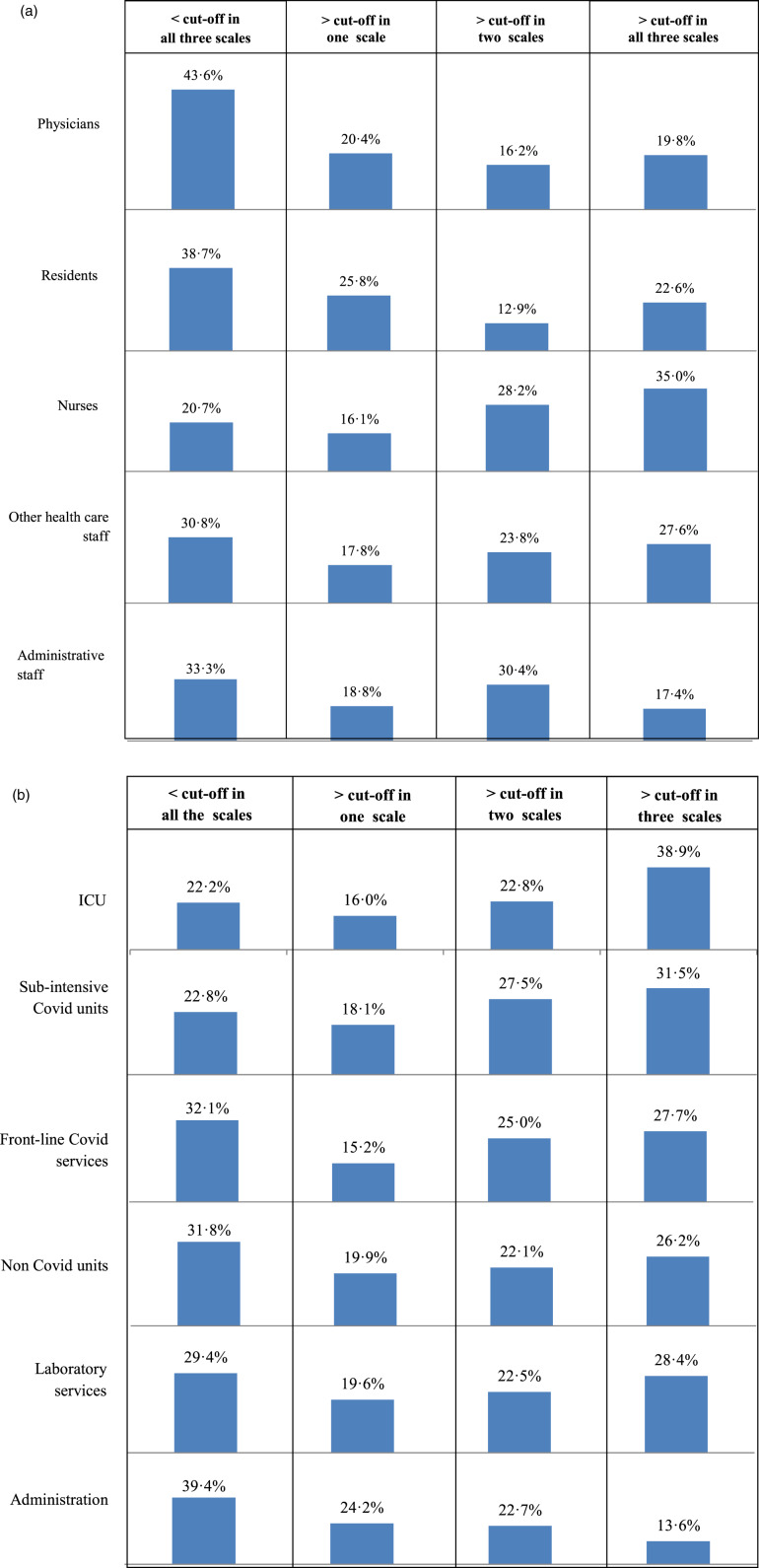
a. Percentage of subjects reporting scores a) < the cut-off in the tree assessment measures; b) scores> cut-off in one assessment measure; c) scores > cut off in two assessment measures; d) scores > cut-off in all the three assessment measures by occupation. Figure 1b. Percentage of subjects reporting scores a) < the cut-off in the tree assessment measures; b) scores> cut-off in one assessment measure; c) scores > cut off in two assessment measures; d) scores > cut-off in all the three assessment measures by place of work.

When computing the percentage of participants scoring above the cut-off point in all the three outcome scales by occupational profile and place of work, the highest proportions were found, respectively, among nurses (35%) (Fig. 1*a*) and healthcare staff working in ICUs (39%) (Fig. 1*b*).

### Risk factors of adverse mental health outcomes

Table 4 shows the unadjusted odds ratios (ORs) estimating the association between each adverse outcome (post-traumatic distress, anxiety and depression) and each potential risk factor.

**Table 4.** Univariable logistic regression models for post-traumatic distress (IES-R ⩾ 24), anxiety (SAS ⩾ 36) and depression (PHQ-9 ⩾ 10) (*n* = 2195)

	Post-traumatic distress		Anxiety		Depression	
	Unadjusted OR (95% CI)	p-value	Unadjusted OR (95% CI)	p-value	Unadjusted OR (95% CI)	p-value
**Gender**						
Male	1		1		1	
Female	1·61 (1·23–2·09)	<0·001	2·56 (2·07–3·17)	<0·001	1·92 (1·49–2·48)	<0·001
**Living condition**						
With family/other relatives	1		1		1	
Alone	1·26 (0·94–1·68)	0·122	1·06 (0·83–1·34)	0·650	1·74 (1·35–2·23)	<0·001
**Work place**						
Intensive care units	1		1		1	
Sub-intensive care wards for COVID-19 pts^a^	0·98 (0·61–1·56)	0·924	0·91 (0·59–1·41)	0·679	0·80 (0·52–1·23)	0·303
Frontline services dealing with COVID-19 pts^b^	0·57 (0·35–0·92)	0·022	0·61 (0·39–0·95)	0·029	0·53 (0·33–0·85)	0·008
Non-COVID wards	0·52 (0·36–0·75)	<0·001	0·51 (0·37–0·71)	<0·001	0·45 (0·33–0·62)	<0·001
Laboratory diagnostic services^c^	0·66 (0·40–1·09)	0·106	0·51 (0·34–0·76)	0·001	0·46 (0·30–0·70)	<0·001
Administration	0·28 (0·16–0·52)	<0·001	0·46 (0·30–0·71)	<0·001	0·31 (0·19–0·51)	<0·001
**Length of working experience**						
<6 yrs	1		1		1	
6–20 yrs	1·30 (0·98–1·72)	0·067	1·25 (1·00–1·57)	0·051	1·08 (0·83–1·39)	0·575
>20 yrs	1·78 (1·35–2·35)	<0·001	1·23 (0·99–1·52)	0·060	1·00 (0·78–1·27)	0·981
**Occupation**						
Physicians	1		1		1	
Residents	0·77 (0·50–1·17)	0·224	1·26 (0·91–1·75)	0·168	1·51 (1·02–2·23)	0·040
Nurses	2·48 (1·74–3·53)	<0·001	3·35 (2·51–4·47)	<0·001	2·26 (1·61–3·18)	<0·001
Other healthcare staff	1·55 (1·06–2·27)	0·024	2·14 (1·58–2·90)	<0·001	1·75 (1·21–2·51)	0·003
Administrative staff	1·27 (0·73–2·22)	0·391	1·65 (1·13–2·40)	0·009	1·21 (0·76–1·93)	0·414
**Having pre-existing psychological problems**						
No	1	–	1	–	1	–
Yes	2.99 (1.76–5.06)	<0.001	2.47 (1.66–3.66)	<0.001	2.69 (1.86–3.89)	<0.001
**Experienced traumatic event**						
No	–	–	1			
Yes	–	–	3·25 (2·69–3·93)	<0·001	3·05 (2·42–3·85)	<0·001
**Afraid of falling ill with COVID-19**						
No	1		1		1	
Yes	2·52 (1·68–3·79)	<0·001	3·60 (2·71–4·79)	<0·001	2·08 (1·49–2·91)	<0·001
Don't know	1·77 (0·82–3·78)	0·143	2·32 (1·32–4·07)	0·003	1·49 (0·76–2·92)	0·250

a Infectious Disease Unit, Pulmonary Medicine, Internal Medicine units converted specifically to COVID-19.

b Radiology and Emergency Department.

c Laboratory Medicine, Transfusion Medicine, Immunology, Pathology, Microbiology.

Table 5 reports multivariable analyses. Adjusted ORs showed that living alone, having a work experience longer than 20 years, being a nurse, having pre-existing psychological problems, having experienced a fear of getting infected with COVID-19 are associated with increased risk of developing severe symptoms of post-traumatic distress; whereas, working in hospital wards/units/offices non-directly engaged in the diagnosis and treatment of COVID patients appeared to be a protective factor concerning those who work in ICUs or sub-intensive COVID units.

**Table 5.** Multivariable logistic regression models for post-traumatic distress (IES-R ⩾ 24), anxiety (SAS ⩾ 36) and depression (PHQ-9 ⩾ 10) (*n* = 2195)

	Post-traumatic distress			Anxiety			Depression		
		*p* value			*p* value			*p* value	
	Adjusted OR (95% CI)	Category	Overall LR test	Adjusted OR (95% CI)	Category	Overall LR test	Adjusted OR (95% CI)	Category	Overall LR test
**Gender**									
Male	1		0·053	1		<0·001	1		<0·001
Female	1·34 (1·00–1·81)	0·053		2·18 (1·71–2·78)	<0·001		1·70 (1·28–2·26)	<0·001	
**Living condition**									
With family/other relatives	1		0·005	1		0·613	1		<0·001
Alone	1·58 (1·15–2·18)	0·005		1·07 (0·82–1·40)	0·613		1·70 (1·29–2·24)	<0·001	
**Work place**									
Intensive care units	1		<0·001	1		0·101	1		0·011
Sub-intensive care wards for COVID-19 pts^a^	0·82 (0·49–1·35)	0·431		0·73 (0·45–1·17)	0·190		0·69 (0·44–1·10)	0·118	
Frontline services dealing with COVID-19 pts^b^	0·47 (0·28–0·79)	0·005		0·70 (0·43–1·15)	0·158		0·58 (0·35–0·95)	0·030	
Non-COVID wards	0·40 (0·27–0·60)	<0·001		0·59 (0·41–0·85)	0·005		0·52 (0·36–0·75)	<0·001	
Laboratory diagnostic services^c^	0·52 (0·29–0·94)	0·030		0·68 (0·42–1·10)	0·114		0·54 (0·33–0·89)	0·015	
Administration	0·24 (0·11–0·51)	<0·001		0·74 (0·42–1·30)	0·294		0·41 (0·22–0·76)	0·005	
**Length of working experience**									
<6 yrs	1		0·017	1		0·685	1		0·478
6–20 yrs	1·12 (0·78–1·60)	0·538		1·15 (0·84–1·56)	0·385		1·22 (0·88–1·70)	0·236	
>20 yrs	1·58 (1·10–2·26)	0·013		1·09 (0·81–1·48)	0·560		1·18 (0·85–1·64)	0·321	
**Occupation**									
Physicians	1		<0·001	1		<0·001	1		0·161
Residents	0·74 (0·43–1·27)	0·269		1·29 (0·83–2·01)	0·256		1·52 (0·92–2·50)	0·099	
Nurses	2·06 (1·40–3·04)	<0·001		2·27 (1·64–3·14)	<0·001		1·59 (1·09–2·31)	0·015	
Other healthcare staff	1·45 (0·94–2·22)	0·089		1·70 (1·20–2·41)	0·003		1·56 (1·04–2·34)	0·030	
Administrative staff	1·58 (0·75–3·31)	0·227		1·51 (0·92–2·50)	0·104		1·54 (0·86–2·76)	0·150	
**Having pre-existing psychological problems**									
No	1	–	<0.001	1	–	<0.001	1	–	<0.001
Yes	2.63 (1.51–4.58)	0.001		2.71 (1.73–4.24)	<0.001		2.61 (1.75–3.89)	<0.001	
**Experienced traumatic event**									
No	–	–		1		<0·001	1		<0·001
Yes	–	–		2·63 (2·12–3·26)	<0·001		2·45 (1·90–3·17)	<0·001	
**Afraid of falling ill with COVID-19**									
No	1		<0·001	1		<0·001	1		0·012
Yes	2·40 (1·55–3·73)	<0·001		2·94 (2·15–4·03)	<0·001		1·69 (1·18–2·43)	0·004	
Don't know	1·45 (0·64–3·30)	0·369		2·00 (1·08–3·69)	0·027		1·47 (0·72–2·99)	0·285	
**Number of observations**	1212			1938			1926		
**LR test, *p* value**	Chi2(16) = 138·31, <0·001			Chi2(17) = 339·20, <0·001			Chi2(17) = 187·82, <0·001		
**Hosmer−Lemeshow goodness-of-fit (10 groups) Chi2(8), *p* value**	4.57, 0·802			4.15, 0·843			14.62, 0·067		
**Pearson goodness-of-fit Number of covariate patterns Chi2(df), *p* value**	306Chi2(289) = 312·61, 0·162			536Chi2(518) = 540·91, 0·235			531Chi2(513) = 559·46, 0·076		
**Area under ROC curve**	0·689			0·729			0·697		

a Infectious Disease Unit, Pulmonary Medicine, Internal Medicine units converted specifically to COVID-19; .

b Radiology and Emergency Department;.

c Laboratory Medicine, Transfusion Medicine, Immunology, Pathology, Microbiology.

With respect to anxiety, being a woman, a nurse or other healthcare staff, having experienced a traumatic event related to COVID-19, having pre-existing psychological problems, and having perceived fear of getting infected are associated with an increased risk of developing symptoms, whereas staff working in non-COVID units display a reduced risk with respect to those working in ICUs.

An increased risk of developing depression was associated with being female, living alone, being a nurse or other healthcare worker, having experienced a traumatic event related to COVID-19, having pre-existing psychological problems, and having perceived fear of getting infected, whereas, working in hospital wards/units/office non-directly engaged in the diagnosis and treatment of COVID-19 patients appeared to be a protective factor compared with those working in ICUs or sub-intensive COVID units.

Overall, being a nurse increased the risk of developing symptoms of post-traumatic distress and anxiety by at least two times with respect to physicians. Having experienced a COVID-related traumatic event increased the risk of developing anxiety and depression by at least 2.5 times. Moreover, having pre-existing psychological problems that required specialised treatment increased the risk of developing post-traumatic distress, anxiety and depression by more than 2.5 times. Finally, the fear of getting infected by COVID-19 increased the risk of developing symptoms of post-traumatic distress by at least two times and the risk of developing anxiety by nearly three times.

Multiple imputation (MI) analysis for missing data was performed for all regression models. Variables included in the MI were occupation, exposure to COVID-19 patients and having pre-existing psychological problems, all significantly associated with response/no response pattern for the outcome measures. All the three variables included in the MI had no missing value (*n* = 2195). Overall, the regression estimates from the complete case and MI analyses are overlapping for all models (details in the on-line supplementary part 5).

Regression models were re-estimated by using an alternative modelling strategy, i.e. treating outcomes as continuous scores. Sensitivity analysis showed that the results of the regressions did not significantly change using an alternative modelling strategy (see the on-line supplementary part 6).

## Discussion

Overall, we found that the great majority of healthcare workers of the Verona University Hospital participating in this survey (86%) reported increased levels of stress at work during the lockdown phase of the COVID-19 outbreak. The pandemic emergency may have determined stressful job conditions for healthcare workers in a number of ways, given their sudden reassignment to other hospital units or new unfamiliar tasks and working under increased workload conditions. Conflict between co-workers was also intensified by the circumstances and might have contributed to increased perception of job-related stress. However, the fear of being infected by COVID-19 has played the most relevant role in determining high levels of perceived stress at work. Studies conducted during the previous epidemics reported increased levels of job-related stress among healthcare workers, whose main concerns regarded their own health and the fear of infecting their families, friends and colleagues; moreover, social isolation, uncertainty, and reluctance to work were reported as other important concerns (Barello *et al*., 2020).

Our survey revealed that a considerable proportion of participants had clinically significant psychological problems, in terms of post-traumatic stress symptoms (54%), anxiety (50%) and depressive symptoms (27%). It is likely that most healthcare workers of the Verona University Hospital developed psychopathological symptoms as a direct response to the COVID-19 pandemic, since only 6% had pre-existing psychological problems. This suggests that the COVID-19 pandemic might have significantly contributed to the development of adverse psychopathological outcomes in the population addressed here. Incidentally, the percentage of healthcare workers with pre-existing psychological problems found in our sample substantially overlaps with prevalence of anxiety and depression found in the Italian general population (Faravelli *et al*., 2004; de Girolamo *et al*., 2006).

We also found that staff working within ICUs or sub-intensive COVID-19 units had a significantly increased risk of developing adverse psychological outcomes (more specifically, post-traumatic distress symptoms and depression), independent of any other factor and that nurses had a considerably greater risk of adverse psychological outcomes (more specifically, post-traumatic distress symptoms and anxiety) than physicians. It is notably that 35% of nurses scored above the cut-off scores in all the three psychological dimensions, thus reflecting a critical mental health condition that deserves timely and careful clinical attention, in terms of services delivered by healthcare professionals, such as counselling or psychotherapy (and, where appropriate, pharmacological treatment). Nurses represent a particularly vulnerable population, as consistently shown by studies carried out during the SARS outbreak (Maunder *et al*., 2004; Wong *et al*., 2005) and the current pandemic (Pappa *et al*., 2020; Song *et al*., 2020). Nurses face a greater risk of exposure to COVID-19 as they spend more time on wards, provide direct care to patients, and have a crucial role in assisting them with all daycare activities. Moreover, due to their closer contact with patients, they may be more exposed to moral injury pertaining to suffering (Williamson *et al*., 2020), death and ethical dilemmas (Robert *et al*., 2020). In addition, the relatively poor involvement in the decision-making process may lead nurses to a passive role that reduces the sense of self-efficacy and increases the perception of worry (Karanikola *et al*., 2014). We also found that healthcare workers suffering from pre-existing psychological problems were particularly vulnerable to the adverse psychological outcomes of the pandemic. This suggests that at the beginning of any epidemic emergency screening for ongoing psychological problems among healthcare workers should be carefully carried out by hospital administration to protect the more vulnerable ones.

Studies conducted during previous epidemics consistently highlight that a relevant proportion of healthcare workers is at risk for developing post-traumatic stress symptoms (Carmassi *et al*., 2020). The development of post-traumatic distress among healthcare workers represents a crucial issue, since the impact of traumatic experiences may have significant long-lasting effects (Dutheil *et al*., 2020). The percentage of staff with symptoms of post-traumatic distress in our study (63%) is higher than that reported by two Chinese studies conducted, respectively, in Tongji Hospital in Wuhan (30%) (Zhu *et al*., 2020) and in People's Hospital in Fuyang City (27%) (Huang *et al*., 2020). The relatively lower morbidity found in these two studies may be explained by the specific psychological protective measures implemented by Tongji Hospital (Zhu et al., 2020) and the inclusion of only physicians in Fuyang City (Huang *et al*., 2020). Prevalence of post-traumatic distress in our study was considerably higher than that found by Song *et al*. (2020) in China, with only 9% showing symptoms of post-traumatic distress. This finding may be related to the timing of the study, as it was conducted in a period during which the pandemic in China had been controlled and the work pressure of the medical staff was significantly reduced. On the other hand, the percentage of post-traumatic distress found in our study is lower than that found by Lai *et al*. (2020) in China, where 70% of respondents had symptoms of post-traumatic distress. The sample of Lai *et al*. (2020), however, was only composed of physicians and nurses working in 41% of cases in COVID-19 front-line services, whereas our sample addressed a broad array of hospital workers, with an overall proportion of front-line staff of 24%. If comparison is restricted to staff working in COVID-19 units only, the proportion of post-traumatic distress found in our study (65%) substantially overlaps with Lai *et al*. (2020).

Regarding anxiety, the proportion of healthcare workers in our study showing clinically significant anxiety symptoms (50%) is far greater than the pooled prevalence of 23% reported by the meta-analysis of Pappa *et al*. (2020). In studies that used the same our assessment measure (Guo et al., 2020; Huang *et al*., 2020; Liu *et al*., 2020a, b) the pooled prevalence was only 16.5%. We may speculate that the higher anxiety morbidity found in our study may be related to the different cut-off scores used to define levels of clinically significant anxiety or to different assessment scales used. However, we have used an internationally validated cut-off score to define clinically significant anxiety (Dunstan and Scott, 2020). Moreover, the difference in morbidity levels is too large to be simply explained by a measurement artefact. We may thus hypothesise that real differences do exist in the emotional response to the pandemic between Italian and Chinese healthcare workers. It is possible that Italian healthcare workers perceived more anxiety since they have never faced an epidemic before, or even received training for managing this kind of emergencies, whereas healthcare workers in China − based on their previous experience with SARS and H1N1- might have been more ready to tackle the current pandemic with greater confidence and less uncertainty.

Regarding depressive symptoms, we found a prevalence of 27%. This finding is consistent with literature, as the pooled prevalence of depressive symptoms among healthcare workers during the COVID-19 pandemic was 23% (Pappa *et al*., 2020). However, three studies (Lai *et al*., 2020; Zhang *et al*., 2020a, b; Zhu *et al*., 2020) using the same scale we used in our survey provided a pooled prevalence of 37%; this inconsistency may be related to methodological issues as two of these studies (Lai *et al*., 2020; Zhang *et al*., 2020a, b) adopted a lower cut-off score. On the other hand, the prevalence of depressive symptoms of our study overlaps with that found in a survey among physicians and nurses (25%) in China (Song *et al*., 2020), and the percentage of ICUs staff with symptoms of depression found in our study (41%) substantially overlaps with that found among frontline staff (45.4%) in China (Zhou *et al*., 2020). Consistent with Kisely *et al*. (2020) and Pappa *et al*. (2020), we also found that the prevalence of depressive symptoms was higher in females [thus probably reflecting the well-known gender gap (Alonso *et al*., 2004)] and in nurses. This latter finding does not reflect the gender gap existing among this professional group, since the effect remained significant also in multivariable analysis that was adjusted for gender.

### Strengths and limitations

The relevance of our findings should be viewed in the light of the fact that: (a) Italy was the first major western country to be affected by the COVID-19 pandemic (and since then one of the most affected countries in the world) and the first in the western world that had to face the related healthcare emergency WorldMeter, 2020); (b) Veneto, within Italy, was the region (together with Lombardy) where the first COVID-19 outbreak was registered and since then has been one of the most affected Italian regions (Ministero della Salute, 2020; Regione Veneto, 2020); (c) the province of Verona was the most burdened area within Veneto during the lockdown period (11 March−4 May 2020), both in terms of deaths and infected cases (Regione Veneto, 2020). Other strengths of this study include the multidimensional examination of psychological outcomes, the large hospital population working in a critical geographical area, the wide array of occupational profiles considered, as well as the ability to collect data, given extreme time constrains and the difficulties associated with conducting research amidst a pandemic. Among the possible limitations, it should be mentioned that the relatively low response rate was 37%. However, web-based surveys have generally lower response rates than face-to-face or telephone interviews or mail surveys; a meta-analysis reported a mean response rate similar to our study (39.6%) (Cook *et al*., 2000). In addition, surveys involving physicians have even lower overall response rates (35%) (Cunningham *et al*., 2015). Another limitation to be considered is that highly distressed healthcare workers, who are generally less likely to engage in surveys or other forms of psychological interventions, might be underrepresented in the study (Kang *et al*., 2020).

### Implications for practice and future research

It is necessary that healthcare systems around the world carefully address the psychological well-being of healthcare workers by, e.g. active monitoring reactions and performance, altering assignments and schedules, modifying expectations, assessing occupational risks and offering − where necessary − psychosocial support (Pfefferbaum and North, 2020). A rapid response team in crisis situations including mental healthcare professionals should be established within each COVID-19 frontline hospital (Kang *et al*., 2020). The type of intervention to provide depends on the stage of the pandemic and the specific mental healthcare needs, ranging from peer support to professional aid. Among the first kind of interventions, a modified version of the ‘Anticipate, Plan, and Deter’ responder risk and resilience model (Schreiber *et al*., 2019) seems to be promising (Albott *et al*., 2020). Among professional interventions, novel therapy approaches such mindfulness, relaxation therapies, EMDR protocols provided through telemental health platforms seem to have a promising role (Bassan *et al*., 2020; Xiao *et al*., 2020; Yang *et al*., 2020). In order to prevent the development of psychological disorders in the long term, active monitoring and provision of psychological support should be delivered also once the crisis begins to recede (Greenberg *et al*., 2020).

Future longitudinal research is needed to evaluate the psychological impact of the pandemic on healthcare workers over the medium- and long-term period and to establish patterns and co-occurrence of risk factors for adverse mental health outcomes. Intervention studies in real-world settings should be additionally conducted and implemented to investigate under which interventions and specific circumstances resilience may be best fostered and the mental health of frontline healthcare professionals supported during and after a disease outbreak.

## Conclusion

This study provided valuable information for policymakers, health administrators, occupational health and mental health professionals on the psychological impact of the COVID-19 outbreak on healthcare staff working in a highly burdened geographical of north-east Italy, which may hopefully assist them in implementing interventions and preventive actions that may be needed in the unfortunate (but highly probable) event of a second pandemic wave.

## Data Availability

Data will be available upon reasonable request.

## Supplementary material

For supplementary material accompanying this paper visit https://doi.org/10.1017/S2045796020001158. click here to view supplementary material
