# Supplementary material for: Psychological impact of COVID-19 pandemic on healthcare workers in a highly burdened area of north-east Italy
Source: Epidemiol Psychiatr Sci. 2019 Dec 17;30:e1. doi: 10.1017/S2045796020001158 (PMC7804082; doi:10.1017/S2045796020001158)
Supplement: Supplementary file 1 [file S2045796020001158sup001.docx]

**The web-survey representativeness (on line part 1)**

The web-survey representativeness refers to the similarity between the response set of the survey compared to the target population (see Figure 1).

**Figure 1.** Target population (Healthcare and administrative staff working in the Verona University Hospital during the lockdown phase of the CIVID-19 pandemic) and web-survey sample

Target population

N=5942

Web-survey sample

N=2195

The response rate was 36·9%. In western countries, web-based surveys have generally lower response rates than face-to-face or telephone interviews or mail surveys: in one meta-analyses, the mean response rate for 68 web-based surveys was 39·6%, similar to the current study.^1^ In addition, it has been found that surveys involving physicians have lower overall response rates (35%) and that specific response rates widely vary according to specialty, ranging from 46% for neurology/neurosurgery to 29% for pediatrics and 27% for psychiatry.^2^

The representativeness of the sample was performed by comparing two key characteristics of participants (occupation and exposure to COVID-19 patients) which have been found associated to response/non response pattern for all three outcome assessments (see on line supplementary) and are available as official statistics of the Verona University Hospital. Exposure to COVID-19 patients has been operationalized by the Hospital as workers employed in wards/units directly engaged with COVID-19 patients due to the re-organization of hospital activities during the pandemic.

By considering these two key-characteristics, the study sample substantially overlap with the overall Verona University Hospital staff (Table 1).

**Table 1.** Occupation and workers employed in wards/units directly engaged with COVID-19 patients for target population and web-survey sample

|  | **Target population**  **N=5942** | | **Web-survey sample**  **N=2195** | |  |
| --- | --- | --- | --- | --- | --- |
|  | **N** | **%** | **N** | **%** | **p-value**  **Chi-square or Fisher’s exact test** |
| **Occupation** |  |  |  |  |  |
| Physicians | 833 | 14·0 | 306 | 13·9 | 0·086 |
| Residents | 1083 | 18·2 | 361 | 16·4 |  |
| Nurses | 2022 | 34·0 | 783 | 35·7 |  |
| Other health care staff | 1509 | 25·4 | 533 | 24·3 |  |
| Administrative staff | 495 | 8·3 | 212 | 9·7 |  |
| **Wards/units directly engaged with COVID-19 patients** |  |  |  |  |  |
| Number of workers | 1134 | 19·1 | 428^§^ | 19·5 | 0·680 |

^§^ Intensive Care Units (n=195) plus Sub-intensive care wards for Covid-19 (n=178) plus 30% Frontline ward/services dealing also with COVID-19 patients (i.e. Radiology) (n=55)

The key characteristics seem to indicate that the respondent selection due to web-survey design did not produce a biased sample with respect to two important indicators in the context of the burden due to COVID-19 pandemic. In the light of this, we are reasonably confident that the sample addressed here may be considered representative of the eligible hospital population.

**References**

1. Cook C, Health F, Thompson R. A Meta-Analysis of Response Rates in Web or Internet-Based Survey. Educ Psychol Measurement 2000; 60: 821-36.

2. Cunningham CT, Quan H, Hemmelgarn B, et al. Exploring physician specialist response rates to web-based surveys. BMC Med Res Methodol 2015; 15: 32.

**Distribution of Job stress (4 items) and Perception of risk (3 items) by work place and occupation (on line part 2)**

**Table 1.** Job stress and perception of risk by work place in the overall sample (n=2195)

|  | **WORK PLACE** (39 missing) | | | | | |
| --- | --- | --- | --- | --- | --- | --- |
|  | **Intensive Care Units** | **Sub-intensive care wards for Covid-19 pts** | **Frontline wards/services dealing with Covid-19 pts** | **Non-COVID wards** | **Laboratory diagnostic services** | **Administration** |
|  | **N (%)** | **N (%)** | **N (%)** | **N (%)** | **N (%)** | **N (%)** |
| **JOB STRESS** (34 missing) |  |  |  |  |  |  |
| **There was more conflict among colleagues***** |  |  |  |  |  |  |
| Yes | 88 (15·2) | 77 (7·2) | 80 (7·5) | 603 (56·2) | 141 (13·1) | 84 (7·8) |
| No | 74 (15·2) | 70 (14·3) | 42 (8·6) | 240 (49·2) | 32 (6·6) | 30 (6·1) |
| As usual | 32 (5·7) | 29 (5·2) | 42 (7·5) | 331 (58·9) | 63 (11·2) | 65 (11·6) |
| **I felt more stressed at work** |  |  |  |  |  |  |
| Yes | 176 (9·7) | 158 (8·7) | 141 (7·8) | 996 (54·8) | 205 (11·3) | 141 (7·8) |
| No | 9 (7·6) | 8 (6·8) | 10 (8·5) | 68 (57·6) | 11 (9·3) | 12 (10·2) |
| As usual | 9 (4·8) | 10 (5·3) | 13 (6·9) | 110 (58·5) | 20 (10·6) | 26 (13·8) |
| **I had to do work that I usually don’t do***** |  |  |  |  |  |  |
| Yes | 155 (12·4) | 134 (10·7) | 108 (8·6) | 638 (50·9) | 124 (9·9) | 95 (7·6) |
| No | 39 (4·5) | 42 (4·8) | 56 (6·4) | 536 (61·7) | 112 (12·9) | 84 (9·7) |
| **I had an increased workload***** |  |  |  |  |  |  |
| Yes | 178 (12·8) | 161 (11·6) | 113 (8·1) | 697 (50·1) | 130 (9·4) | 111 (8·0) |
| No | 16 (2·2) | 15 (2·0) | 51 (7·0) | 477 (65·1) | 106 (14·5) | 68 (9·3) |
| **PERCEPTION OF RISK** (34 missing) |  |  |  |  |  |  |
| **I should not be looking after patients with COVID-19**  (796 subjects do not look after COVID-19 pts)*** |  |  |  |  |  |  |
| No | 147 (16·6) | 124 (14·0) | 117 (13·2) | 440 (49·8) | 34 (3·8) | 22 (2·5) |
| Yes | 39 (8·5) | 43 (9·4) | 30 (6·6) | 284 (62·1) | 35 (7·7) | 26 (5·7) |
| **I accept the risk of getting COVID-19 as part of my job** (566 subject do not face with COVID-19 pts)** |  |  |  |  |  |  |
| No | 19 (12·3) | 14 (9·0) | 11 (7·1) | 87 (56·1) | 8 (5·2) | 16 (10·3) |
| Yes | 171 (12·1) | 154 (10·9) | 142 (10·1) | 779 (55·2) | 113 (8·0) | 53 (3·8) |
| **I’m afraid of getting ill with COVID-19***^#^** |  |  |  |  |  |  |
| No | 15 (5·0) | 21 (7·0) | 15 (5·0) | 170 (57·0) | 46 (15·4) | 31 (10·4) |
| Yes | 174 (9·9) | 148 (8·4) | 146 (8·3) | 979 (55·8) | 176 (10·0) | 133 (7·6) |
| Don’t know | 5 (7·2) | 7 (10·1) | 3 (4·3) | 25 (36·2) | 14 (20·3) | 15 (21·7) |

*** p<0·001 ** p<0·01 ^#^ 1 cell has frequency <5

**Table 2.** Job stress and perception of risk by occupation in the overall sample (n=2195)

|  | **OCCUPATION** | | | | |
| --- | --- | --- | --- | --- | --- |
|  | **Physicians** | **Residents** | **Nurses** | **Other health care staff** | **Administrative staff** |
|  | **N (%)** | **N (%)** | **N (%)** | **N (%)** | **N (%)** |
| **JOB STRESS** (34 missing) |  |  |  |  |  |
| **There was more conflict among colleagues***** |  |  |  |  |  |
| Yes | 142 (13·0) | 181 (16·6) | 397 (36·4) | 281 (25·8) | 90 (8·2) |
| No | 68 (13·8) | 92 (18·6) | 195 (39·5) | 103 (20·9) | 36 (7·3) |
| As usual | 93 (16·1) | 83 (14·4) | 180 (31·3) | 138 (24·0) | 82 (14·2) |
| **I felt more stressed at work***** |  |  |  |  |  |
| Yes | 242 (13·1) | 277 (15·0) | 704 (38·1) | 460 (24·9) | 164 (8·9) |
| No | 21 (17·5) | 37 (30·8) | 26 (21·7) | 23 (19·2) | 13 (10·8) |
| As usual | 40 (20·6) | 42 (21·6) | 42 (21·6) | 39 (20·1) | 31 (16·0) |
| **I had to do work that I usually don’t do***** |  |  |  |  |  |
| Yes | 151 (11·9) | 253 (19·9) | 506 (39·8) | 275 (21·6) | 87 (6·8) |
| No | 152 (17·1) | 103 (11·6) | 266 (29·9) | 247 (27·8) | 121 (13·6) |
| **I had an increased workload***** |  |  |  |  |  |
| Yes | 151 (10·7) | 181 (12·8) | 618 (43·7) | 354 (25·0) | 111 (7·8) |
| No | 152 (20·4) | 175 (23·5) | 154 (20·6) | 168 (22·5) | 97 (13·0) |
| **PERCEPTION OF RISK** (34 missing) |  |  |  |  |  |
| **I should not be looking after patients with COVID-19**  (796 subjects do not look after COVID-19 pts)*** |  |  |  |  |  |
| No | 139 (15·4) | 161 (17·9) | 393 (43·7) | 180 (20·0) | 27 (3·0) |
| Yes | 44 (9·5) | 51 (11·0) | 233 (50·1) | 112 (24·1) | 25 (5·4) |
| **I accept the risk of getting COVID-19 as part of my job** (566 subject do not face with COVID-19 pts)*** |  |  |  |  |  |
| No | 9 (5·7) | 11 (7·0) | 79 (50·0) | 36 (22·8) | 23 (14·6) |
| Yes | 226 (15·7) | 261 (18·2) | 593 (41·3) | 310 (21·6) | 47 (3·3) |
| **I’m afraid of getting ill with COVID-19***** |  |  |  |  |  |
| No | 57 (19·0) | 73 (24·3) | 79 (26·3) | 64 (21·3) | 27 (9·0) |
| Yes | 240 (13·4) | 274 (15·3) | 673 (37·6) | 437 (24·4) | 166 (9·3) |
| Don’t know | 6 (8·5) | 9 (12·7) | 20 (28·2) | 21 (29·6) | 15 (21·1) |

*** p<0·001

**Differences between completers and non-completers on assessments (on line part 3)**

In Figure 1 is given the flow-chart representing how assessments have been completed by the participants (n=2195).

**Figure 1.** Flow-chart of assessments for the whole sample (n=2195)

Experienced traumatic event

No

N=791

Yes

N=1361

Missing

N=43

IES-R

N=1361

Yes

N=1242

No

N=119

SAS

N=2195

PHQ-9

N=2195

Yes

N=1991

No

N=204

Yes

N=1979

No

N=216

Consent to participate in the web-survey

N=2195

Personal information

N=2195

Occupation, having pre-existing psychological problems, exposure to COVID-19 patients

N=2195

Living condition

N=2188

Gender, age

N=2186

Length of working experience

N=2181

Work place

N=2156

Perceived job stress (4 items), perception of risk (3 items)

N=2161

Personal information showed negligible percentages of missing values (from 0% for occupation, ongoing treatment for psychological problems and exposure to COVID-19 patients to 1·8% for work place) mainly due to a very limited number of participants who did not want to be recognisable. Ad hoc schedules exploring perceived job stress and perception of risk were not completed by 1·5% of participants.

By considering the outcome assessments, IES-R was not completed by 119 participants out of 1361 (8·7%), SAS by 204 out of 2195 (9·3%), and PHQ by 216 out of 2195 (9·8%).

The differences between completers and non-completers for each outcome assessment were explored with respect to personal information (Table 1) and job stress and perception of risk (Table 2).

Occupation and exposure to CODIV-19 patients were associated with the pattern of compilation for all three assessments. IES-R response/no response was also associated to gender, work place and the item ‘I had to do work that I usually don’t do’; SAS to have experienced traumatic events and the item ‘I accept the risk of getting COVID-19 as part of my job’; PHQ-9 showed the same associations of SAS, but it was also associated to ‘I had to do work that I usually don’t do’.

Finally, the 43 participants who did not give information about to have experienced traumatic event were compared, with respect to personal information and the two schedules job stress and perception of risk, to the 2152 who gave this information. No characteristic resulted significantly associated with the pattern of response/no response, with the only exception of exposure to COVID-19 patients (p<0·01), for which the percentages of those who gave a response about the traumatic event were distributed, with respect to the categories of exposure to COVID, as follows: 97·1% for Rarely/Never, 99·1% for Daily/Some days a week and 97·6% for Don’t know.

**Table 1.** Personal information for completers and non-completers (n=2195)

|  | **IES-R**  **N (%)** | | **SAS**  **N (%)** | | **PHQ-9**  **N (%)** | |
| --- | --- | --- | --- | --- | --- | --- |
|  | **Completers**  **N=1242** | **Non-completers**  **N=119** | **Completers**  **N=1991** | **Non-completers**  **N=204** | **Completers**  **N=1979** | **Non-completers**  **N=216** |
| **Gender** |  |  |  |  |  |  |
| Male | 288 (94·1)* | 18 (5·9)* | 500 (92·8) | 39 (7·2) | 496 (92·0) | 43 (8·0) |
| Female | 951 (90·5)* | 100 (9·5)* | 1484 (90·1) | 163 (9·9) | 1476 (89·6) | 171 (10·4) |
| **Age** |  |  |  |  |  |  |
| <36 yrs | 417 (92·9) | 32 (7·1) | 644 (91·7) | 58 (8·3) | 640 (91·2) | 62 (8·8) |
| 36-55 yrs | 641 (91·1) | 63 (8·9) | 998 (90·6) | 104 (9·4) | 990 (89·8) | 112 (10·2) |
| >55 yrs | 181 (88·7) | 23 (11·3) | 344 (90·1) | 38 (9·9) | 344 (90·1) | 38 (9·9) |
| **Living condition** |  |  |  |  |  |  |
| Alone | 228 (93·8) | 15 (6·2) | 329 (91·9) | 29 (8·1) | 324 (90·5) | 34 (9·5) |
| With family/other relatives | 1010 (90·7) | 103 (9·3) | 1656 (90·5) | 174 (9·5) | 1649 (90·1) | 181 (9·9) |
| **Occupation** |  |  |  |  |  |  |
| Physicians | 169 (94·9)** | 9 (5·1)** | 290 (94·8)** | 16 (5·2)** | 289 (94·4)** | 17 (5·6)** |
| Residents | 190 (95·0)** | 10 (5·0)** | 338 (93·6)** | 23 (6·4)** | 336 (93·1)** | 25 (6·9)** |
| Nurses | 514 (89·9)** | 58 (10·1)** | 698 (89·1)** | 85 (10·9)** | 695 (88·8)** | 88 (11·2)** |
| Other health care staff | 297 (91·1)** | 29 (8·9)** | 473 (88·7)** | 60 (11·3)** | 470 (88·2)** | 63 (11·8)** |
| Administrative staff | 72 (84·7)** | 13 (15·3)** | 192 (90·6)** | 20 (9·4)** | 189 (89·2)** | 23 (10·8)** |
| **Work place** |  |  |  |  |  |  |
| Intensive Care Units | 163 (94·8)** | 9 (5·2)** | 184 (94·4) | 11 (5·6) | 183 (93·8) | 12 (6·2) |
| Sub-intensive care wards for Covid-19 | 152 (95·6)** | 7 (4·4)** | 166 (93·3) | 12 (6·7) | 166 (93·3) | 12 (6·7) |
| Frontline wards/services dealing with Covid-19 | 119 (94·4)** | 7 (5·6)** | 149 (90·9) | 15 (9·1) | 146 (89·0) | 18 (11·0) |
| Non-COVID wards | 618 (90·1)** | 68 (9·9)** | 1075 (89·7) | 124 (10·3) | 1071 (89·3) | 128 (10·7) |
| Laboratory diagnostic services | 104 (90·4)** | 11 (9·6)** | 221 (92·9) | 17 (7·1) | 220 (92·4) | 18 (7·6) |
| Administration | 68 (84·0)** | 13 (16·0)** | 163 (89·6) | 19 (10·4) | 160 (87·9) | 22 (12·1) |
| **Length of working experience** |  |  |  |  |  |  |
| <6 yrs | 382 (92·3) | 32 (7·7) | 605 (90·6) | 63 (9·4) | 601 (90·0) | 67 (10·0) |
| 6-20 yrs | 395 (90·8) | 40 (9·2) | 603 (90·0) | 67 (10·0) | 595 (88·8) | 75 (11·2) |
| >20 yrs | 457 (90·9) | 46 (9·1) | 770 (91·3) | 73 (8·7) | 770 (91·3) | 73 (8·7) |
| **Exposure to Covid-19 patients** |  |  |  |  |  |  |
| Daily/Some days a week | 726 (94·0)*** | 46 (6·0)*** | 880 (92·4)** | 72 (7·6)** | 876 (92·0)** | 76 (8·0)** |
| Rarely/Never | 336 (85·9)*** | 55 (14·1)*** | 724 (88·4)** | 95 (11·6)** | 720 (87·9)** | 99 (12·1)** |
| Don’t know | 180 (90·9)*** | 18 (9·1)*** | 387 (91·3)** | 37 (8·7)** | 383 (90·3)** | 41 (9·7)** |
| **Having pre-existing psychological problems** |  |  |  |  |  |  |
| Yes | 81 (92·0) | 7 (8·0) | 124 (91·9) | 11 (8·1) | 124 (91·9) | 11 (8·1) |
| No | 1161 (91·2) | 112 (8·8) | 1867 (90·6) | 193 (9·4) | 1855 (90·0) | 205 (10·0) |
| **Experienced traumatic event** |  |  |  |  |  |  |
| Yes | 1242 (91·3) | 119 (8·7) | 1217 (89·4)*** | 144 (10·6)*** | 1211 (89·0)*** | 150 (11·0)*** |
| No | - | - | 774 (97·9)*** | 17 (2·1)*** | 768 (97·1)*** | 23 (2·9)*** |

Chi-square or Fisher’s exact test, where appropriate * p<0·05 **p<0·01 ***p<0·001

**Table 2.** Job stress and perception of risk for completers and non-completers (n=2195)

|  | **IES-R**  **N (%)** | | **SAS**  **N (%)** | | **PHQ-9**  **N (%)** | |
| --- | --- | --- | --- | --- | --- | --- |
|  | **Completers**  **N=1242** | **Non-completers**  **N=119** | **Completers**  **N=1991** | **Non-completers**  **N=204** | **Completers**  **N=1979** | **Non-completers**  **N=216** |
| **JOB STRESS** |  |  |  |  |  |  |
| **There was more conflict among colleagues** |  |  |  |  |  |  |
| Yes | 685 (91·1) | 67 (8·9) | 1002 (91·8) | 89 (8·2) | 999 (91·6) | 92 (8·4) |
| No | 290 (93·9) | 19 (6·1) | 460 (93·1) | 34 (6·9) | 457 (92·5) | 37 (7·5) |
| As usual | 267 (89·0) | 33 (11·0) | 529 (91·8) | 47 (8·2) | 523 (90·8) | 53 (9·2) |
| **I felt more stressed at work** |  |  |  |  |  |  |
| Yes | 1136 (91·4) | 107 (8·6) | 1700 (92·0) | 147 (8·0) | 1694 (91·7) | 153 (8·3) |
| No | 43 (89·6) | 5 (10·4) | 111 (92·5) | 9 (7·5) | 107 (89·2) | 13 (10·8) |
| As usual | 63 (90·0) | 7 (10·0) | 180 (92·8) | 14 (7·2) | 178 (91·8) | 16 (8·2) |
| **I had to do work that I usually don’t do** |  |  |  |  |  |  |
| Yes | 838 (93·2)*** | 61 (6·8)*** | 1183 (93·0) | 89 (7·0) | 1178 (92·6)* | 94 (7·4)* |
| No | 404 (87·4)*** | 58 (12·6)*** | 808 (90·9) | 81 (9·1) | 801 (90·1)* | 88 (9·9)* |
| **I had an increased workload** |  |  |  |  |  |  |
| Yes | 962 (91·5) | 89 (8·5) | 1296 (91·6) | 119 (8·4) | 1290 (91·2) | 125 (8·8) |
| No | 280 (90·3) | 30 (9·7) | 695 (93·2) | 51 (6·8) | 689 (92·4) | 57 (7·6) |
| **PERCEPTION OF RISK** |  |  |  |  |  |  |
| **I should not be looking after patients with COVID-19** (796 NA) |  |  |  |  |  |  |
| No | 616 (92·4) | 51 (7·6) | 830 (92·2) | 70 (7·8) | 825 (91·7) | 75 (8·3) |
| Yes | 328 (92·1) | 28 (7·9) | 424 (91·2) | 41 (8·8) | 421 (90·5) | 44 (9·5) |
| **I accept the risk of getting COVID-19 as part of my job** (566 NA) |  |  |  |  |  |  |
| No | 108 (88·5) | 14 (11·5) | 137 (86·7)** | 21 (13·3)** | 136 (86·1)** | 22 (13·9)** |
| Yes | 929 (92·7) | 73 (7·3) | 1331 (92·6)** | 106 (7·4)** | 1324 (92·1)** | 113 (7·9)** |
| **I’m afraid of getting ill with COVID-19** |  |  |  |  |  |  |
| No | 113 (91·1) | 11 (8·9) | 283 (94·3) | 17 (5·7) | 279 (93·0) | 21 (7·0) |
| Yes | 1093 (91·4) | 103 (8·6) | 1644 (91·8) | 146 (8·2) | 1637 (91·5) | 153 (8·5) |
| Don’t know | 36 (87·8) | 5 (12·2) | 64 (90·1) | 7 (9·9) | 63 (88·7) | 8 (11·3) |

Chi-square or Fisher’s exact test, where appropriate * p<0·05 **p<0·01 ***p<0·001

**Reported traumatic events (on line part 4)**

Participants who declared to have experienced a traumatic event during COVID-19 pandemic (n=1361) were asked to describe it in a free text field. Unstructured data in the form of text were found to be an extremely rich source of information. After a first reading of answers, FA and AL proposed a text classification by assigning categories according to text content. Then, FA and AL independently assigned all descriptions to one of these categories. When the process was terminated, they compared their assignment and achieved a consensus whenever they disagreed.

Figure 1 shows the results of this text classification.

**Figure 1.** Frequency distribution of reported traumatic events (n=1361)

A = Fear to be infected and/or to infect others

B = Feeling under pressure due to time and staff constraints

C = Having to deal with high number of deaths in a relatively short time, to see patients dying alone as relatives were not allowed to enter the restricted areas or to communicate by telephone the death of beloved one to relatives

D = Working with insufficient supervision, unclear treatment guidelines/protocols, shortage of personal protective equipments

E = Having been reassigned to COVID-19 units or to unfamiliar tasks without a sufficient/adequate notice and/or adequate training

F = Difficulty of balancing work and family life

G = Having received notice of infection or death of a relative, friend or colleague

H = Having to make difficult ethical decisions in a short time

I = Having realized to be infected with COVID-19

**Multiple Imputation for outcome missing values (on line part 5)**

Variables included in the multiple imputation (MI) analysis were occupation and exposure to COVID-19 patients, both significantly associated to response/no response pattern for all three outcome measures. Moreover, having pre-existing psychological problems was added to the analysis because it had a strong correlation with incomplete outcomes. All the three variables included in the MI analysis had no missing value (n=2195).

Imputation package for implementation was Stata 15 (command ‘mi impute logit’ with M=20 imputations). After that, regression models for the outcomes were re-estimated by the command ‘mi estimate, or: logit’.

For each model, the following parameters were given: number of observations; average RVI (Relative Increase in Variance), that is an increase in the variance of the estimate because of the loss of information about the parameter due to nonresponse relative to the variance of the estimate with no information lost - the closer to zero, the less effect missing data have on the variance of the estimate; largest FMI (Fraction of Missing Information), that is the largest of all the FMI about coefficient estimates due to nonresponse - a rule of thumb is that M ≥ 100*FMI provides an adequate level of reproducibility of MI analysis; the model F test to reject the hypothesis that all ORs are equal to one.

**Imputation of IES-R**

Number of observations: complete 1242, imputed 119, total 1361

The results of the multivariable logistic regression models for IES-R after MI are given in Table 1.

**Table 1.** Multivariable logistic regression model for post-traumatic distress (IES-R) (n=2195)

|  | **Post-traumatic distress** | |
| --- | --- | --- |
|  | **Adjusted OR**  **(95% CI)** | **p-value** |
| **Gender** |  |  |
| Male | 1 |  |
| Female | 1·33 (0.99-1·79) | 0·059 |
| **Living condition** |  |  |
| With family/Other people | 1 |  |
| Alone | 1·54 (1·12-2·13) | 0·008 |
| **Work place** |  |  |
| Intensive Care Units | 1 |  |
| Sub-intensive COVID units | 0·84 (0·51-1·39) | 0·495 |
| Frontline services dealing with COVID | 0·49 (0·29-0·82) | 0·007 |
| Non COVID units | 0·42 (0·28-0·62) | <0·001 |
| Laboratory diagnostic services | 0·52 (0·29-0·92) | 0·026 |
| Administration | 0·27 (0·13-0·57) | 0·001 |
| **Length of working experience** |  |  |
| <6 yrs | 1 |  |
| 6-20 yrs | 1·13 (0·79-1·61) | 0·512 |
| >20 yrs | 1·56 (1·09-2·25) | 0·016 |
| **Occupation** |  |  |
| Physician | 1 |  |
| Resident | 0·73 (0·43-1·26) | 0·264 |
| Nurse | 2·07 (1·41-3·02) | <0·001 |
| Other health care staff | 1·48 (0·97-2·26) | 0·071 |
| Administrative staff | 1·49 (0·73-3·05) | 0·272 |
| **Having pre-existing psychological problems** |  |  |
| No | 1 |  |
| Yes | 2.54 (1.45-4.44) | 0.001 |
| **Experienced traumatic events** |  |  |
| No | - | - |
| Yes | - | - |
| **Afraid of falling ill with COVID** |  |  |
| No | 1 |  |
| Yes | 2·17 (1·41-3·36) | <0·001 |
| Don’t know | 1·40 (0·62-3·20) | 0·420 |
| **Number of observations** | 1324 |  |
| **Average RVI** | 0·107 |  |
| **Largest FMI** | 0·151 |  |
| **F (df1, df2)**  **p-value** | F (16, 34904) = 6·76  <0·001 |  |

**Imputation of SAS and PHQ-9**

Number of observations for SAS: complete 1991, imputed 204, total 2195

Number of observations for PHQ-9: complete 1979, imputed 216, total 2195

The results of the multivariable logistic regression models for SAS and PHQ-9 after MI are given in Table 2.

**Table 2.** Multivariable logistic regression model for anxiety (SAS) and depression (PHQ-9) (n=2195)

|  | **Anxiety** | | **Depression** | |
| --- | --- | --- | --- | --- |
|  | **Adjusted OR**  **(95% CI)** | **p-value** | **Adjusted OR**  **(95% CI)** | **p-value** |
| **Gender** |  |  |  |  |
| Male | 1 |  | 1 |  |
| Female | 2·06 (1·62-2·62) | <0·001 | 1·62 (1·22-2·15) | 0·001 |
| **Living condition** |  |  |  |  |
| With family/Other people | 1 |  | 1 |  |
| Alone | 1·07 (0·82-1·39) | 0·623 | 1·64 (1·25-2·16) | <0·001 |
| **Work place** |  |  |  |  |
| Intensive Care Units | 1 |  | 1 |  |
| Sub-intensive COVID units | 0·75 (0·47-1·20) | 0·224 | 0·68 (0·43-1·07) | 0·095 |
| Frontline services dealing with COVID | 0·72 (0·44-1·18) | 0·199 | 0·58 (0·35-0·95) | 0·031 |
| Non COVID units | 0·59 (0·41-0·85) | 0·005 | 0·52 (0·36-0·74) | <0·001 |
| Laboratory diagnostic services | 0·67 (0·42-1·09) | 0·106 | 0·52 (0·32-0·86) | 0·011 |
| Administration | 0·75 (0·43-1·31) | 0·307 | 0·41 (0·22-0·75) | 0·004 |
| **Length of working experience** |  |  |  |  |
| <6 yrs | 1 |  | 1 |  |
| 6-20 yrs | 1·16 (0·85-1·57) | 0·351 | 1·21 (0·86-1·68) | 0·269 |
| >20 yrs | 1·10 (0·81-1·48) | 0·553 | 1·16 (0·84-1·61) | 0·361 |
| **Occupation** |  |  |  |  |
| Physician | 1 |  | 1 |  |
| Resident | 1·27 (0·82-1·97) | 0·289 | 1·49 (0·90-2·46) | 0·118 |
| Nurse | 2·27 (1·64-3·14) | <0·001 | 1·62 (1·11-2·36) | 0·012 |
| Other health care staff | 1·71 (1·21-2·41) | 0·002 | 1·58 (1·06-2·37) | 0·026 |
| Administrative staff | 1·46 (0·88-2·43) | 0·146 | 1·44 (0·81-2·55) | 0·214 |
| **Having pre-existing psychological problems** |  |  |  |  |
| No | 1 |  | 1 |  |
| Yes | 2.70 (1.71-4.24) | <0.001 | 2.64 (1.79-3.91) | <0.001 |
| **Experienced traumatic events** |  |  |  |  |
| No | 1 |  | 1 |  |
| Yes | 2·46 (1.99-3·04) | <0·001 | 2·32 (1·80-2.99) | <0·001 |
| **Afraid of falling ill with COVID** |  |  |  |  |
| No | 1 |  | 1 |  |
| Yes | 2·72 (2.00-3·69) | <0·001 | 1·59 (1·11-2·27) | 0·011 |
| Don’t know | 1·91 (1.02-3·56) | 0·042 | 1·41 (0·70-2·85) | 0·341 |
| **Number of observations** | 2091 |  | 2091 |  |
| **Average RVI** | 0·089 |  | 0·081 |  |
| **Largest FMI** | 0·132 |  | 0·114 |  |
| **F (df1, df2)**  **p-value** | F (17, 44571) = 14·37  <0·001 |  | F (17, 54518) = 8·66  <0·001 |  |

**Conclusions**

For all regression models, the average RVI values range from 0·081 for PHQ-9 to 0·107 for IES-R, while the 20 imputations exceed the required number of imputations according to the FMI rule.

The regression estimates from complete case (CC) and multiple imputation (MI) analyses are overlapping for all models.

**Sensitivity analysis (on line part 6)**

Outcome regression models have been re-estimated by using an alternative modelling strategy, that is treating outcomes as continuous scores.

Table 1 shows the multivariable linear regression models for total IES-R, SAS and PHQ-9, respectively.

Direction and statistical significance of the results of linear and logistic modelling are very similar for all the outcomes, with some negligible differences.

**Table 1.** Multivariable linear regression models for post-traumatic distress (IES-R total score), anxiety (SAS total score) and depression (PHQ-9 total score) (n=2195)

|  | **Post-traumatic distress** | | **Anxiety** | | **Depression** | |
| --- | --- | --- | --- | --- | --- | --- |
|  | **Adjusted Beta (95% CI)** | **p-value** | **Adjusted Beta (95% CI)** | **p-value** | **Adjusted Beta (95% CI)** | **p-value** |
| **Gender** |  |  |  |  |  |  |
| Male | Ref. |  | Ref. |  | Ref. |  |
| Female | 3·75 (1·38, 6·13) | 0·002 | 3·28 (2·36, 4·20) | <0·001 | 1·39 (0·89, 1·90) | <0·001 |
| **Living condition** |  |  |  |  |  |  |
| With family/Other people | Ref. |  | Ref. |  | Ref. |  |
| Alone | 3·06 (0·58, 5·53) | 0·016 | 0·42 (-0·60, 1·44) | 0·421 | 0·89 (0·33, 1·45) | 0·002 |
| **Work place** |  |  |  |  |  |  |
| Intensive Care Units | Ref. |  | Ref. |  | Ref. |  |
| Sub-intensive COVID units | -0·68 (-4·48, 3·12) | 0·725 | -0·84 (-2·63, 0.95) | 0·359 | -0·58 (-1·56, 0·39) | 0·243 |
| Frontline services dealing with COVID | -6·69 (-10·79, -2·59) | 0·001 | -2·95 (-4·80, -1.10) | 0·002 | -1·35 (-2·36, -0·33) | 0·009 |
| Non COVID units | -8·24 (-11·25, -5.23) | <0·001 | -2·85 (-4.23, -1·47) | <0·001 | -1·18 (-1·93, -0·43) | 0·002 |
| Laboratory diagnostic services | -5·90 (-10·42, -1.38) | 0·011 | -1.37 (-3.18, 0·44) | 0·138 | -0·64 (-1·62, 0·35) | 0·206 |
| Administration | -9·33 (-15·08, -3·59) | 0·001 | -2·35 (-4·48, -0·22) | 0·031 | -1·37 (-2·54, -0·21) | 0·021 |
| **Length of working experience** |  |  |  |  |  |  |
| <6 yrs | Ref. |  | Ref. |  | Ref. |  |
| 6-20 yrs | 1·00 (-1.84, 3·84) | 0·489 | 0·64 (-0·54, 1·83) | 0·289 | 0·65 (0·00, 1·30) | 0·050 |
| >20 yrs | 4·82 (1.98, 7·66) | 0·001 | 0·56 (-0·61, 1·73) | 0·347 | 0·67 (0·03, 1·31) | 0·040 |
| **Occupation** |  |  |  |  |  |  |
| Physician | Ref. |  | Ref. |  | Ref. |  |
| Resident | -1.64 (-5.90, 2·62) | 0·451 | 1·49 (-0·17, 3·15) | 0·079 | 1·77 (0·86, 2·68) | <0·001 |
| Nurse | 5·91 (2.79, 9·03) | <0·001 | 3.93 (2·69, 5·16) | <0·001 | 1·16 (0·49, 1·84) | 0·001 |
| Other health care staff | 3·27 (-0·18, 6·71) | 0·063 | 2·47 (1·15, 3·80) | <0·001 | 0·77 (0·04, 1·49) | 0·038 |
| Administrative staff | 1·72 (-4.06, 7·50) | 0·560 | 1·45 (-0·46, 3·37) | 0·137 | 0·63 (-0·41, 1·68) | 0·237 |
| **Having pre-existing psychological problems** |  |  |  |  |  |  |
| No | Ref. |  | Ref. |  | Ref. |  |
| Yes | 7.75 (3.91, 11.59) | <0.001 | 6.29 (4.74, 7.85) | <0.001 | 2.98 (2.13, 3.83) | <0.001 |
| **Experienced traumatic** |  |  |  |  |  |  |
| No | - | - | Ref. |  | Ref. |  |
| Yes | - | - | 4·52 (3·69, 5·35) | <0·001 | 2·22 (1·77, 2·68) | <0·001 |
| **Afraid of getting COVID** |  |  |  |  |  |  |
| No | Ref. |  | Ref. |  | Ref. |  |
| Yes | 7·98 (4·65, 11·30) | <0·001 | 4·19 (3.09, 5·28) | <0·001 | 1·40 (0·80, 2·00) | <0·001 |
| Don’t know | 5·59 (-0·83, 12.01) | 0·088 | 1·62 (-0·70, 3·94) | 0·171 | 1·14 (-0·14, 2·41) | 0·081 |
| **Number of observations** | 1212 | | 1938 | | 1926 | |
| **F test, p-value** | F(16, 1195)=11·33, <0·001 | | F(17, 1920)=33·06, <0·001 | | F(17, 1908)=19·51, <0·001 | |
| **Adj R-squared** | 12·0% | | 22·0% | | 14·0% | |
